# Is Asian tiger mosquito (*Aedes albopictus*) going to become homodynamic in Southern Europe in the next decades due to climate change?

**DOI:** 10.1098/rsos.220967

**Published:** 2022-12-14

**Authors:** Irene Del Lesto, Claudio De Liberato, Riccardo Casini, Adele Magliano, Arianna Ermenegildi, Federico Romiti

**Affiliations:** ^1^ Department of Grosseto, Istituto Zooprofilattico Sperimentale del Lazio e della Toscana ‘M. Aleandri’, Viale Europa 30, 58100 Grosseto, Italy; ^2^ Istituto Zooprofilattico Sperimentale del Lazio e della Toscana M. Aleandri, Via Appia nuova 1411, 00178, Rome (RM), Italy

**Keywords:** invasive mosquito, Culicidae, disease vector, risk map, global warming, climate change

## Abstract

The Asian tiger mosquito, *Aedes albopictus*, competent vector of several arboviruses, poses significant impact on human health worldwide. Although global warming is a driver of *A. albopictus* range expansion, few studies focused on its effects on homodynamicity (i.e. the ability to breed all-year-round), a key factor of vectorial capacity and a primary condition for an *Aedes*-borne disease to become endemic in temperate areas. Data from a 4-year monitoring network set in Central Italy and records from weather stations were used to assess winter adult activity and weekly minimum temperatures. Winter oviposition occurred in 38 localities with a seasonal mean photoperiod of 9.7 : 14.3 (L : D) h. Positive collections (87) occurred with an average minimum temperature of the two and three weeks before sampling of approximately 4°C. According to these evidences and considering the climate projections of three global climate models and three shared socio-economic pathways for the next three 20-year periods (from 2021 to 2080), the minimum temperature of January will increase enough to allow an all-year-round oviposition of *A. albopictus* in several areas of the Mediterranean Basin. Due to vector homodynamicity, *Aedes*-borne diseases could become endemic in Southern Europe by the end of the twenty-first century, worsening the burden on human health.

## 1. Background

The Asian tiger mosquito *Aedes albopictus* (Skuse 1894), native to Southeast Asia, is an invasive species that in the last 50 years was able to colonize tropical and temperate areas of all continents, except for Antarctica. Accidentally introduced in Europe in the 1970s, it is actually present in more than 20 countries of this continent [1–3]. Arrived in Northern Italy by tyre trade in 1990, *A. albopictus* is nowadays widespread and common in all Italian regions [4–6], mainly in human-made habitats within urban areas [7–11]. Due to the ongoing climate change, its distribution range is expected to further expand towards higher latitudes and altitudes [12–14].

*Aedes albopictus* can be vector of more than 26 arboviruses, ranking second for its relevance as disease vector only to *Aedes aegypti*, among the invasive mosquitoes of the genus *Aedes* [15,16]. Indeed, its global expansion was frequently followed by the occurrence of outbreaks of *Aedes*-borne viral diseases in naive areas [17,18], becoming a serious health concern worldwide [16]. In Europe, this species has been involved in the transmission of dengue and chikungunya viruses during recent outbreaks [19–22], so much that the European Centre for Diseases Prevention and Control (ECDC) stressed the need for a comprehensive understanding of the risk associated with this mosquito [23,24].

In this scenario, many studies focused on the possible geographical spread of *A. albopictus* in temperate regions due to climate change and on the concomitant expansion of the risk of occurrence of *A. albopictus*-borne diseases [10,25]. Cold winter temperatures have been identified as the most limiting factor for *A. albopictus* range expansion in Europe [26,27] and January's minimum temperatures play a crucial role in *A. albopictus* survival [28]. Surprisingly enough, few studies took into consideration another important factor dealing with human health threats posed by *A. albopictus*, i.e. the risk that, due to global warming, this species could in the future become homodynamic in some temperate regions. Monitoring activities carried out in three regions of the Mediterranean Basin showed evidence of winter activity of the species: homodynamicity in Spain [29,30] and oviposition in Italy and Lebanon [31,32]. Should *A. albopictus* became homodynamic in temperate regions in the future due to climate change, the risk of an endemization of viruses such as dengue and chikungunya, once accidentally introduced, could be concrete. Actually, two chikungunya outbreaks occurred in Italy in 2007 and 2017 [33,34], ending with the onset of the cold season, due to the interruption of *A. albopictus* activity.

At least two factors drive the deposition of diapausing eggs and the interruption of adult activity of *A. albopictus* in temperate regions, temperature and photoperiod [35], without a clear picture of the interaction between the two. Previous findings on the species ecology report thresholds of approximately 10°C in temperature and a photoperiod of 13 : 11 (L : D) h as triggers for the onset of egg-laying activity and larval stages development [35–37]. However, photoperiod seems to be less strict in its influence on *A. albopictus* activity, as demonstrated by several reports of winter activity in temperate regions [29–31,35–37].

In this scenario, the present work was carried out in order to predict, according to the principal models of climate change, where and when *A. albopictus* could become homodynamic in Southern Europe and the Mediterranean Basin, highlighting those areas where the species impact on human health might increase.

## 2. Methods

### 2.1. Sampling design

**Figure 1.**
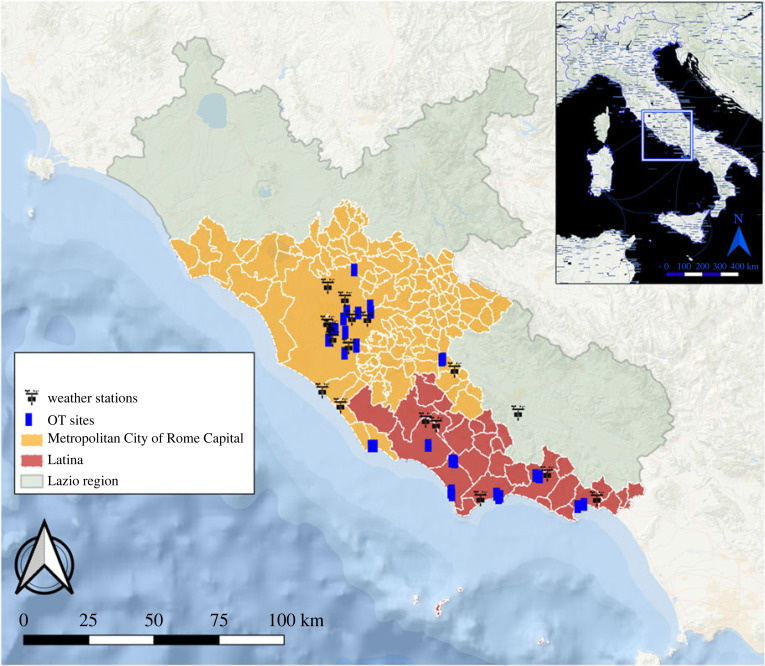
Map of the study area reporting the sampling localities where winter (1 December–28 February) positive OTs were recorded during the study period.

The study was carried out in the Lazio region, Central Italy. Following the chikungunya outbreak that occurred in the southern part of the region in 2017 [34], an *A. albopictus* monitoring network active all-year-round by means of standard ovitraps (OTs) was set [37]. Each OT consisted of 400 ml black plastic container three-quarters filled with tap water and equipped with a Masonite strip (15 × 3 cm) for oviposition. For the present study, a subset of winter positive OTs was selected in the time interval 2018–2022, i.e. those OTs with at least one laid egg in a whole winter season. Winter extent was defined as beginning in December and ending in February (1 December–28 February) [38]. This selection yielded a main dataset, consisting of 38 positive sites, for a total of 87 positive collections, within 12 municipalities in the province of Latina and Metropolitan City of Rome Capital (table 1, figure 1). A secondary dataset was created, considering only the oviposition activity recorded after the 21 December (winter solstice), in order to exclude an egg-laying activity linked to an autumn elongated phenology. The secondary dataset, consisting in 29 OT sites and 46 positive collections, was used to calculate the average of minimum temperatures of the two and three weeks before each positive collection, to identify winter minimum temperatures permissive for sporadic egg-laying activity.

**Table 1.** Summary of the sampling activity carried out during four consecutive winter seasons. The total number of samplings and active OTs of the monitoring network in each winter season (WMN) were reported together with the total number of samplings and active OTs of the main dataset (MD). Number of MD's positive samplings and positive OTs was reported along with their percentage calculated on MD. The mean eggs count per positive OT was reported for MD and the secondary dataset (SD).

sampling season (period)	WMN	MD			mean eggs per positive OT (±*σ*)	
	samplings no./active OTs no.	samplings no./active OTs no.	positive samplings no. (%)	positive OTs no. (%)	MD	SD
first winter (Dec 2018–Feb 2019)	185/52	88/19	8 (9.1)	8 (42.1)	9.5 (± 15.4)	17.0 (± 25.1)
second winter (Dec 2019–Feb 2020)	129/36	87/25	24 (27.6)	20 (80.0)	15.9 (± 23.0)	22.4 (± 25.8)
third winter (Dec 2020–Feb 2021)	146/38	115/28	31 (27.0)	17 (60.7)	15.8 (± 31.8)	5.1 (± 9.6)
fourth winter (Dec 2020–Feb 2021)	263/47	179/31	24 (13.4)	15 (48.4)	6.9 (± 8.9)	3.7 (± 3.3)

WMN, winter monitoring network; OT, ovitrap; MD, main dataset (OTs from 1 December to 28 February); SD, secondary dataset (OTs from 21 December to 28 February); *σ*, standard deviation.

OTs were placed in each municipality outdoor, at ground level, in sheltered and shaded places, and were left in the same position throughout the whole study period. OTs were set in urban areas, within public or house gardens, hospitals or seats of the local health service, gathering places (e.g. markets, train stations and churches), graveyards and container terminals. The Masonite strips were collected fortnightly and eggs were counted under a stereomicroscope. For further details about the sampling design and *A. albopictus* eggs identification refer to Romiti *et al.* [37].

### 2.2. Weather and climate data

To obtain the average values of January minimum temperature (*J*_min_), raster data of minimum temperatures of the coldest month were obtained from WorldClim website (https://www.worldclim.org/data/index.html). WorldClim provides 19 bioclimatic variables (BIO) at global scale and at 30 arcsec resolution (approx. 1 km^2^). Among these, BIO6, which is a single-layer raster file representing the average *J*_min_ over the time interval 1970–2000, was used to characterize the coldest month's temperatures of the recent past in the Mediterranean area. A second set of WorldClim variables, the historical monthly minimum temperatures (HM_min_), was downloaded to obtain the average of *J*_min_ of each year over a 58-year period, from 1961 to 2018 of each OT site (spatial resolution 2.5 arcmin, approx. 21 km^2^). HM_min_ were analysed together with weather station data to characterize temperature changes, from the recent past to the present, in the study area (see *Data analysis*). Similarly, future projections of the BIO variables (19 f-BIO) were downloaded at 2.5 arcmin resolution (approx. 21 km^2^) to obtain the predicted *J*_min_ (f-BIO6) of each OT site. Future BIO variables were available from WorldClim according to 23 global climate models (GCMs) and to four shared socio-economic pathways (SSPs): 1–2.6, 2–4.5, 3–7.0 and 5–8.5 [39]. We considered three GCMs, namely the Canadian Earth System Model v. 5 (CanESM5) [40], the Hadley Centre Global Environment Model v. 3 (HadGEM3) [41] and the Model for Interdisciplinary Research on Climate v. 6 (MIROC6) [42], from the Intergovernmental Panel on Climate Change sixth assessment (CMIP6). The choice of these three GCMs was based on their performance in describing precipitations and surface air temperatures above the European region [43]. The f-BIO were downloaded as averages over three 20-year periods: 2021–2040, 2041–2060 and 2061–2080, and under three CMIP6 SSPs, namely SSP 1–2.6, SSP 3–7.0 and SSP 5–8.5. The three SSPs selected were chosen to provide a range of distinct end-of-century climate change scenarios, from the optimistic SSP 1–2.6 to the ‘business as usual’ scenario (SSP 5–8.5), which does not enact any climate policies. According to these SSPs, the mean warming at the end of the century will be 2.0°C (SSP 1–2.6), 4.1°C (SSP 3–7.0) or 5.0°C (SSP 5–8.5). For HadGEM3, the medium stabilization scenario SSP 3–7.0 was not available, so the SSP 2–4.5 was used instead (warming limit by the end of twenty-first century = 3°C). BIO6, HM_min_ and f-BIO6 were also used to produce, respectively, recent past, current and future scenarios of *J*_min_ in Southern Europe and the Mediterranean Basin.

Climatic data relative to the sampling seasons (2018–2022) were obtained from 17 thermo-pluviometric weather stations in the provinces of Latina, Frosinone and Metropolitan City of Rome Capital, freely available from the LineaMeteo network (https://retemeteo.lineameteo.it/index.php) (figure 1). Considering the importance of the minimum temperatures on the onset of egg-laying activity, daily minimum temperatures, over the four winter seasons, were obtained from the nearest weather station to each OT site, with available weather data (mean distance = 10.26 km, maximum distance = 34.15 km, minimum distance = 0.58 km).

### 2.3. Data analysis

The historical averages of *J*_min_ (1961–2018) of each OT in the main dataset were obtained from HM_min_ using the extract function in the raster R package [44]. The monthly average of *J*_min_ during the sampling period (2019–2022) was calculated for each OT site from daily minimum temperatures, recorded by the weather stations network. The whole monthly *J*_min_ (1961–2022) were used to analyse the changes in January minimum temperatures from the recent past to the present.

Weather stations data were used to calculate the average of minimum temperatures of the two and three weeks before the collection date of each OT in the secondary dataset (positive OTs between 21 December and 28 February) for each winter season. Daily mean rainfall and hours of light (photoperiod) were also calculated using, respectively, the weather stations data and the daylength function in geosphere R package [45].

Future predictions of *J*_min_ (f-BIO6) of each OT in the main dataset were extracted from each GCM (as above described for HM_min_) and according to the three SSPs, over the three 20-year periods. The increasing trend in minimum temperatures was visualized to highlight differences among GCMs/SSPs.

To produce and visualize the recent past, current and future scenarios of *J*_min_ in Southern Europe and the Mediterranean area, we used the QGIS software [46]. The *J*_min_ values from BIO, HM_min_ and f-BIO were divided in three different ranges defined by two thresholds. The first threshold separates temperatures too cold to allow winter oviposition from those permissive for sporadic egg-laying activity. To calculate this limit we averaged the means of minimum temperatures of the two and three weeks before a positive OT collection, considering only the OTs belonging to the secondary dataset. The second threshold separates sporadic oviposition from homodynamicity. This limit was set to 10°C, considering both findings on species ecology and previous reports of homodynamicity in Southern Europe [29,30,35–37].

## 3. Results

### 3.1. General outcomes

**Figure 2.**
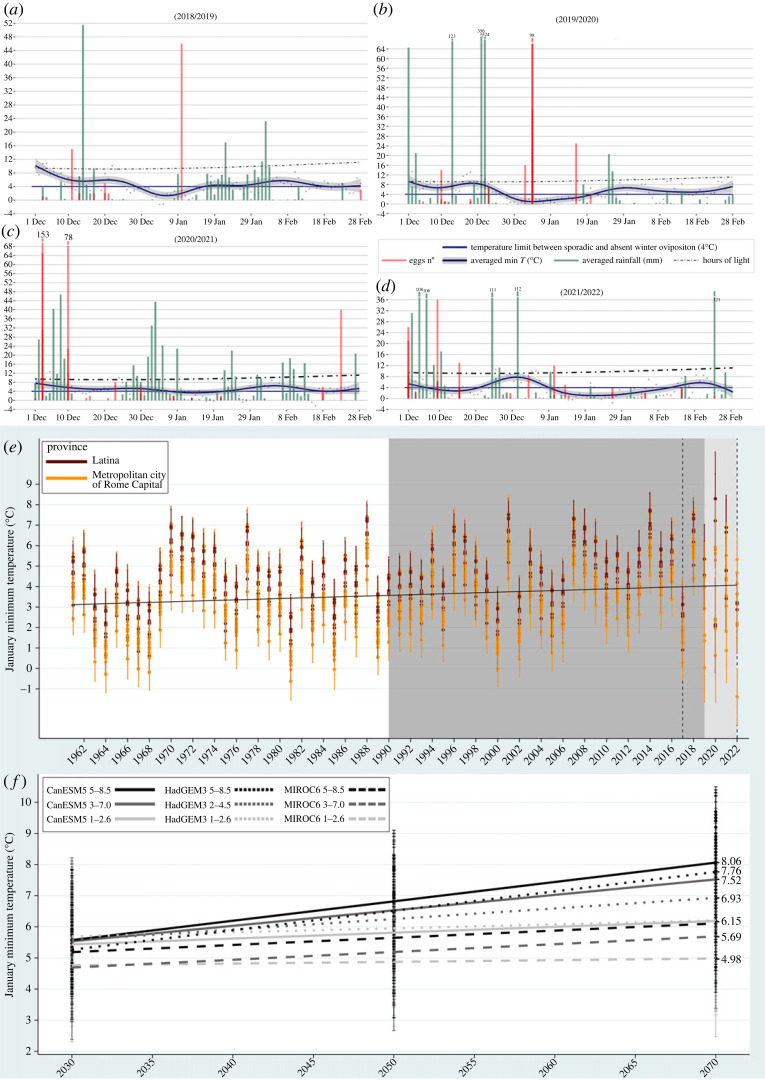
Sampling and weather data of the four winter seasons (*a*–*d*) with daily means of minimum temperatures and rainfalls. In (*e*), the dashed vertical lines indicate the extent of the monitoring network; the dark grey rectangle highlights *A. albopictus* invasion of Italy; the light grey rectangle highlights the averaged minimum temperatures of January (*J*_min_) calculated from weather stations data. In (*f*), the observed trends in increasing *J*_min_ as reported by the nine combination of GCMs/SSPs for all sampling sites.

Overall, 469 samplings were carried out between December and February in four consecutive winter seasons (2018–2022), with a total of 1113 laid eggs, 87 positive OTs and 38 positive sites (mean number of approx. 13 eggs/OT). The mean winter photoperiod of the study area was 9.7 : 14.3 (L : D) h, never exceeding the 12 light hours (figure 2*a–d*). Considering the main dataset, the seasonal mean percentage of positive collections (±*σ*) was 19.1% (±9.6%), with a mean percentage (±*σ*) of 57.8% (±16.7%) positive sites. The mean laid eggs (±*σ*) for the main and the secondary datasets was, respectively, 12.8 (±23.5) and 11.5 (±18.9) (table 1). The overall maximum laid eggs (*n* = 153) was recorded in the Terracina municipality (Latina province), in the first week of December 2020.

A total of 46 positive collections occurred after the winter solstice (secondary dataset): 20 in the Latina province and 26 in the Metropolitan City of Rome. Concerning the secondary dataset, the averaged means of the minimum temperatures in the two and three weeks before each positive sampling were, respectively, 4.25°C and 4.85°C. Thus, the threshold between cold temperatures, which prevent winter oviposition, and those allowing sporadic egg-laying was set to 4°C. As regards the secondary dataset, the highest eggs counts in the two provinces were recorded in the first 10 days of January: 98 eggs in the Metropolitan City of Rome (4 January 2020) and 46 eggs in the Fondi municipality (10 January 2019) (figure 2*a*,*b*). Concurrently, average minimum temperatures of the two and three weeks before sampling were, respectively, 3.07°C and 4.62°C (Rome) and 4.41°C and 5.99°C (Fondi).

### 3.2. Past and current scenarios

In 58 years (1961–2018), the average *J*_min_ increased from 3°C to 4°C in the study area, according to HM_min_ dataset (figure 2*e*). According to the weather data from the thermo-pluviometric stations, the warming trend continued in the last four winter seasons (2019–2022), exceeding 4°C in the study area.

**Figure 3.**
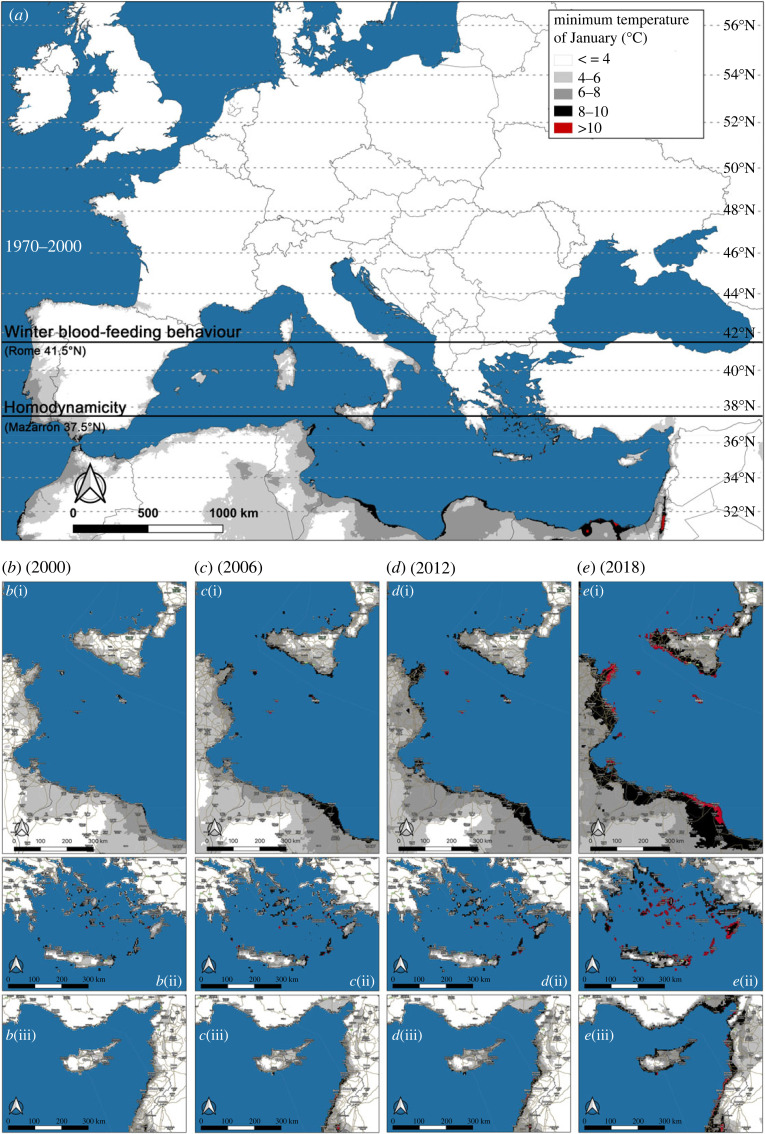
In (*a*), averaged minimum temperatures of January (*J*_min_) in the recent past (BIO database from WorldClim). Black horizontal lines indicate latitudes where homodynamicity (37.5°N) and winter blood-feeding behaviour (41.5°N) have been recorded. In (*b*–*e*), increasing *J*_min_ values over the first two decades of the twenty-first century (HM_min_ database from WorldClim), focusing on: (i) Southern Italian, Tunisian and Western Libyan shorelines; (ii) Greek islands of the southern portion of the Aegean Sea; (iii) Eastern Mediterranean Sea with the shorelines of Turkey, Israel, Lebanon, Syria and Cyprus island.

The average *J*_min_ of the Mediterranean area, according to BIO6 (average over the period 1970–2000), showed limited areas of the Southern Mediterranean shorelines in the range 8–10°C, with spotted localities above 10°C (Dead Sea shores and northern portion of Egypt, figure 3*a*). According to the HM_min_ database and focusing on three regions of the Mediterranean Basin (Sicilian Sea, Greek islands and Cyprus), the coastal zones showed an increasing extent of the areas affected by 8–10°C (figure 3*b*–*e*). Indeed, the shorelines of these regions presented wide areas characterized by a *J*_min_ above 10°C in 2018, particularly evident in Sicily, Tunisia and Greek islands (figure 3*e*(i),(ii)).

### 3.3. Future scenarios

According to the f-BIO database, the *J*_min_ is expected to increase by 1–4°C by the end of the twenty-first century in the study area, considering the GCMs' climate projections of the last analysed 20-year period (2061–2080). All the combinations of GCMs/SSPs consistently described a warming scenario, even if differing in intensity. The most pessimistic model (CanESM5), combined with the ‘business as usual’ SSP (5–8.5), predicted a *J*_min_ of 8°C, whereas according to the most optimistic model (MIROC6), coupled with the high mitigation SSP (1–2.6), *J*_min_ will reach approximately 5°C (figure 2*f*).

**Figure 4.**
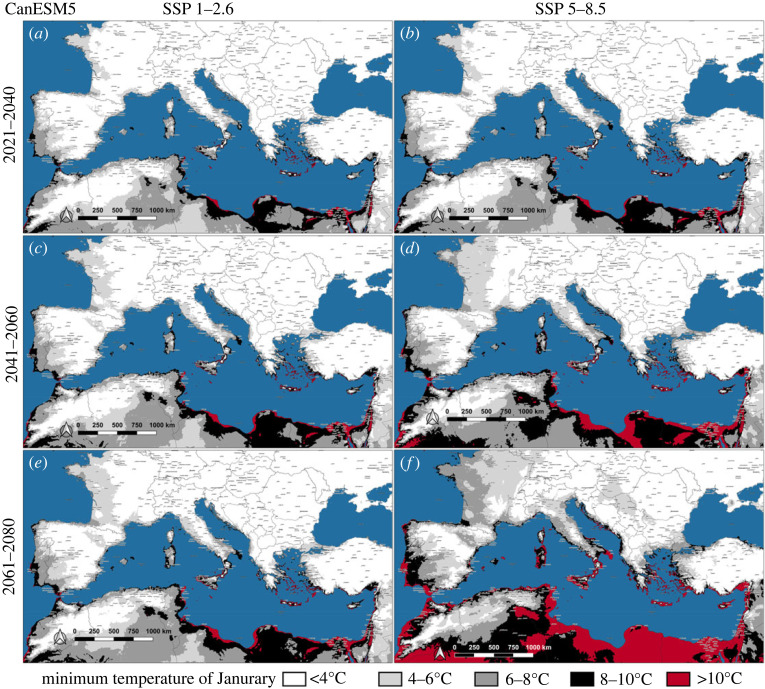
Future scenarios of January minimum temperature (*J*_min_) on the Mediterranean Basin and surrounding areas according to the predictions of CanESM5, coupled with low and high emission pathways (SSP 1–2.6 and SSP 5–8.5).

**Figure 5.**
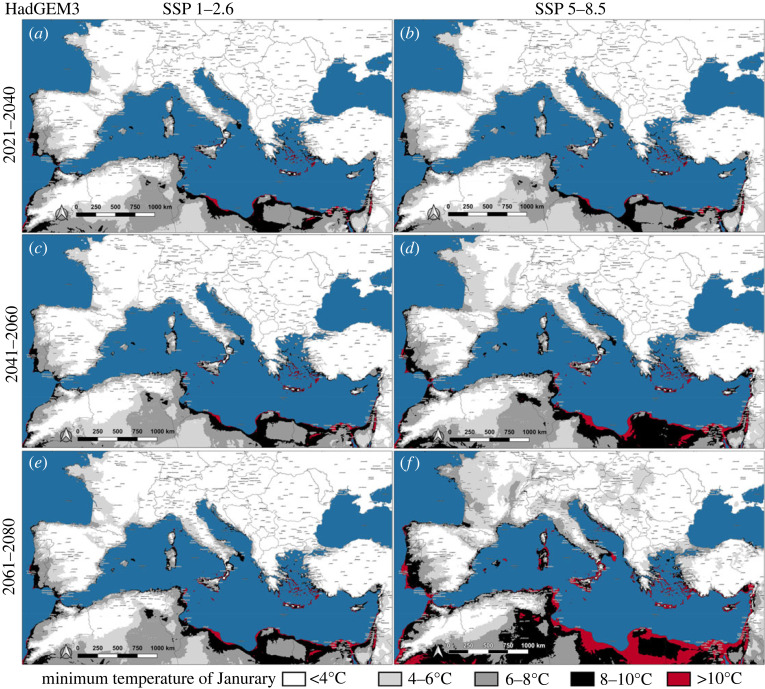
Future scenarios of January minimum temperature (*J*_min_) on the Mediterranean Basin and surrounding areas according to the predictions of HadGEM3, coupled with low and high emission pathways (SSP 1–2.6 and SSP 5–8.5).

**Figure 6.**
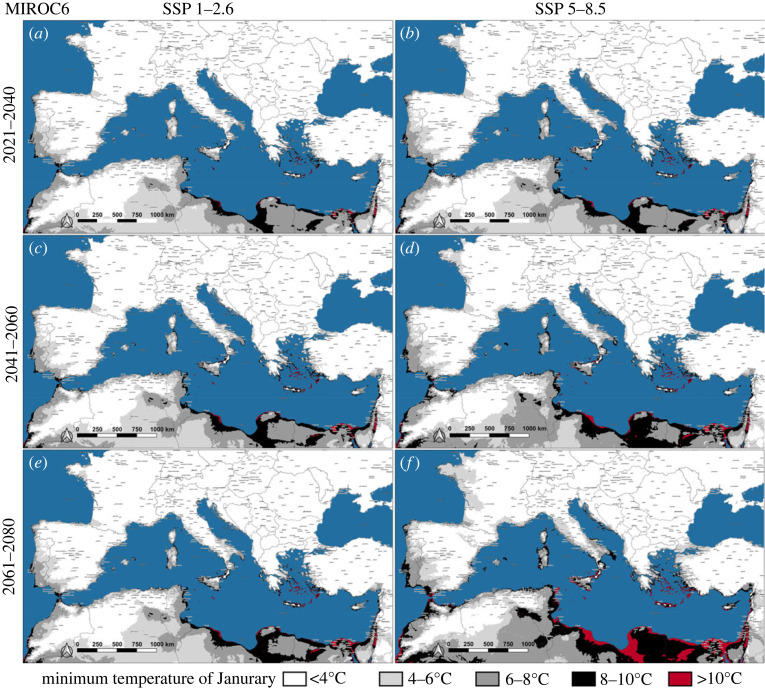
Future scenarios of January minimum temperature (*J*_min_) on the Mediterranean Basin and surrounding areas according to the predictions of MIROC6, coupled with low and high emission pathways (SSP 1–2.6 and SSP 5–8.5).

Maps representing the future *J*_min_ of Southern Europe were produced considering the low and high emissions pathways (i.e. 1–2.6 and 5–8.5) of each GCM (figures 4–6). According to all GCMs, a sporadic winter oviposition in the coastal areas of Southern Europe is predicted to occur in the near future, even considering the high mitigation pathway (figures 4*a*, 5*a* and 6*a*). During the first analysed 20-year period (2021–2040), temperatures in the range 4–10°C (*J*_min_) would be widely distributed along the Mediterranean shorelines, from the Strait of Gibraltar to the eastern portion of the Mediterranean Basin, with the only exception of those lands bordering the Northwestern Adriatic sea. Within this time interval, even according to the most optimistic scenario (MIROC6/1–2.6), large portions of the Iberian peninsula would be affected by *J*_min_ within the range 4–10°C, particularly Central-southern Portugal and the Spanish regions of Extremadura, Murcia and Andalucia, with spotted zones exceeding 10°C (e.g. Province of Cádiz) (figure 6*a*). Regarding the GCMs' scenarios for the last two analysed 20-year periods (2041–2080), the Mediterranean islands between the Strait of Sicily and the Levantine Basin will be affected by a *J*_min_ above 10°C, clearly evident in the CanESM5 and HadGEM3 projections (figures 4*c*–*f* and 5*c*–*f*) and, to a lesser extent, from the MIROC6 estimates (figure 6*c*–*f*). The mainland Europe would not present wide areas characterized by a *J*_min_ above 10°C, apart from Southern Italy, Greece and Atlantic shores of Spain and Portugal (figures 4*f* and 5*f*). Nevertheless, in the second half of the twenty-first century, spotted localities affected by a *J*_min_ within the range 8–10°C will be widespread in Southern Europe, approximately at latitudes below the 43°N, even considering the low emission pathway of all GCMs.

## 4. Discussion

Even if sporadically, *A. albopictus* winter oviposition occurred for four consecutive winter seasons, namely during the whole study period, in several urban areas widespread in Central Italy. As regards the city of Rome, the first findings on winter activity of this species date back to the winter 2003/2004, showing a weekly mean of positive OTs around 30% [31]. Our results showed highest percentages of positive collections during the second and third winter (2019–2020 and 2020–2021), although with lower values than those reported by Romi *et al*. [31]. Unexpectedly, even with an average of minimum temperatures of the two and three weeks before sampling around 4°C, positive OTs were collected, regardless of the short photoperiod. Oviposition implicates, in anautogenous mosquito species, the occurrence of a blood meal [47]. Considering the countries bordering the Mediterranean Basin, egg-laying females, hence blood-feeding behaviour, were already recorded in wintertime in the metropolitan area of Rome (41°53′36″ N, 12°28′58″ E), in the municipality of Mazarrón, Southeastern Spain (37°35′54″ N, 1°18′50″ W) and in two localities near Beirut, Lebanon (mean latitude = 33°45′35″ N) [30–32]. Romi *et al*. [31] formulated two hypotheses to explain the occurrence of winter oviposition: some long-lived females could have continued their trophic activity throughout the winter season, or, alternatively, one or more complete life cycles may have occurred (i.e. homodynamicity). Key component of both hypotheses, from an epidemiological point of view, is the occurrence of trophic activity in winter season, thus entailing a possible epidemiological risk related to *Aedes*-borne diseases, extending to all-year-round the period of arbovirus transmission in Southern Europe. Clear evidence of *A. albopictus* homodynamicity was found in Southern Europe where eggs, larvae and adults were recorded in Southern Spain (approx. 37°N) during winter [29,30]. Photoperiod is considered a critical environmental factor influencing both onset and end of diapausing eggs' oviposition, with 11–11.5 h of daylight needed for the winter eggs to hatch in the field [35–37,48]. Nevertheless, it has been hypothesized that *A. albopictus* populations from Rome could exhibit polymorphic response to photoperiod, with some females that could lay non-diapausing eggs in mild winters [35], a phenomenon that could be the result of the genetic admixture between temperate and tropical *A. albopictus* strains recorded in Rome [49]. It has indeed been demonstrated that life-history traits of temperate *A. albopictus* populations show high plasticity in response to photoperiod, when compared with tropical strains lacking photoperiodic diapause [50,51].

However, further studies are needed to untangle the complex relationship between photoperiod and temperature, as determinants of diapausing eggs production. A great relevance of temperature would be indicated by the homodynamicity recorded in Southern Spain (approx. 37°N), characterized by mild winters and a mean winter photoperiod of 10.1 h of daylight, a daylight duration that, at higher latitudes, is concurrent with a 100% of diapause incidence (43°39′52″ N, Cagnes-sur-Mer, France) [29,52]. Hence, the response to photoperiod could be considered plastic, sensitive to larval nutritional status and temperature, which has been demonstrated to be the most important factor driving diapausing/non-diapausing egg oviposition [35,53]. Such evidence raises particular concerns, since many countries of the Mediterranean Basin, located around 37°N or even below this latitude, could be affected by an *A. albopictus* homodynamicity: e.g. southern portions of Greece, Italy and Turkey, the Northern Algeria and Tunisia and the Republics of Cyprus and Malta. Besides, the extent of those areas characterized by a *J*_min_ consistent with a sporadic winter oviposition (4–10°C) or a continuous *A. albopictus* life cycle (greater than 10°C) clearly increased in the first two decades of the twenty-first century, particularly in the Central-eastern Mediterranean Basin (figure 3*b*–*e*). These areas reached their greatest extension in 2018 (figure 3*e*), the fourth warmest year of the century, characterized by a significant heatwave in Central Europe between late July and early August [54].

By contrast to many model predictions [23,55], which overrated the relevance of rainfall to estimate future *A. albopictus* spread, the species succeeded in the colonization of the arid coastal zones of Southern Europe, mostly relying on rural or peridomestic human-made breeding sites for the water supply [29,34]. Considering the last update of the current European distribution of *A. albopictus* (March 2022), the species is already established in almost all the Mediterranean islands and northern shorelines of the Mediterranean Basin, the only exceptions being Cyprus and Southern Turkey coasts [56]. Namely, the species is nowadays present in all those areas of Southern Europe where mild winters might at present favour homodynamicity (figure 3*e*). As regards future climate, even according to the most optimistic scenario (MIROC6/SSP 1–2.6) (figure 6), by the second half of twenty-first century several coastal areas of Southern Europe would be affected by winter *A. albopictus* trophic activity. Hence, even reducing emissions to low-baseline pathway (+2°C by 2100), the Mediterranean Basin will hold areas possibly affected by a continuous *A. albopictus* life cycle. On the opposite, considering the ‘business as usual’ pathway combined with the worst GCM (CanESM5/SSP 5–8.5) (figure 4), *A. albopictus* homodynamicity would affect wide areas of many Mediterranean countries, where autochthonous vector-borne transmission has already caused outbreaks of chikungunya, dengue and zika: Portugal, Spain, France, Italy, Croatia and Egypt [57]. This would raise a major health concern about the possible endemization of *Aedes*-borne diseases in Southern Europe and the Mediterranean Basin.

The scenarios provided represent a stepping-stone for further researches, mapping areas in the Mediterranean Basin where weather conditions could allow, in the near future, winter activity of the species. Additional studies are needed to take into account other epidemiological factors such as vector abundance and distribution, human population density and extrinsic incubation period. Indeed, modelling and combining these parameters with our results, it would be possible to highlight areas that may be at risk of endemization of *Aedes-*borne diseases.

According to the last statement on global climate, key climatic factors draw a picture of a warming world, where the temperatures are predicted to further increase together with the frequency of extreme meteorological events, such as heatwaves and heavy rainfalls (e.g. Medicane in 2021) [58–61]. Although the proposed results should be taken with due caution by policymakers, prompt mitigation actions, aimed at stabilizing the global average temperature increase, and vector surveillance programmes should be implemented to prevent *Aedes*-borne diseases endemization in areas at risk, especially since a greater impact of weather and climate threats are expected to target the Mediterranean shorelines, concurrently with an increase in human population density along coastal areas [62,63].

## Data Availability

The models on future climate as well as historical climate data used in this paper are licensed under a Creative Commons Attribution-ShareAlike 4.0 International License (https://creativecommons.org/licenses/) and accessible from WorldClim website (https://www.worldclim.org/data/cmip6/cmip6climate.html).
